# Measurement of fractional exhaled nitric oxide and nasal nitric oxide in male patients with obstructive sleep apnea

**DOI:** 10.1007/s11325-018-1760-1

**Published:** 2018-12-12

**Authors:** Dongmei Zhang, Yi Xiao, Jinmei Luo, Xiaona Wang, Yixian Qiao, Rong Huang, Wei Wu

**Affiliations:** 1 Department of Respiratory Medicine, Peking Union Medical College Hospital, Chinese Academy of Medical Sciences & Peking Union Medical College, Beijing, China; 2 Present Address: Department of Respiratory and Critical Care Medicine, Beijing Institute of Respiratory Medicine and Beijing Chaoyang Hospital, Capital Medical University, Beijing, China; 3 Department of Clinical Laboratory, Peking Union Medical College Hospital, Chinese Academy of Medical Sciences & Peking Union Medical College, Beijing, China

**Keywords:** Obstructive sleep apnea, Airway inflammation, Exhaled nitric oxide, Fractional exhaled nitric oxide, Nasal nitric oxide

## Abstract

**Objective:**

Airway inflammation plays an important role in obstructive sleep apnea (OSA); exhaled nitric oxide is regarded as a noninvasive marker of airway inflammation. The aim of this study was to evaluate fractional exhaled nitric oxide (FeNO) and nasal nitric oxide (nNO) in patients with OSA.

**Methods:**

Seventy-five patients with OSA and 30 health controls were enrolled in this study. FeNO and nNO were measured before and after sleep. Nasal lavage was performed in 31 non-smoking individuals immediately after NO measurement in the morning. The sample of nasal lavage was taken for cell classification and analyzing interleukin 6 (IL-6) and interleukin 8 (IL-8).

**Results:**

Both FeNO and nNO were significantly higher in OSA (before sleep FeNO 21.08 ± 8.79 ppb vs.16.90 ± 6.86 ppb, *p* = 0.022; after sleep FeNO 25.57 ± 15.58 ppb vs.18.07 ± 6.25 ppb, *p* = 0.003; before sleep nNO 487.03 ± 115.83 ppb vs. 413.37 ± 73.10 ppb, *p* = 0.001; after sleep nNO 550.07 ± 130.24 ppb vs. 460.43 ± 109.77 ppb, *p* < 0.001). Furthermore, in non-smoking OSA, nNO levels were positively correlated with apnea hypopnea index (AHI) and average decrease of pulse arterial oxygen saturation (SpO_2_); after sleep, nNO was also positively associated to recording time with SpO_2_ < 90% and negatively associated to minimum SpO_2_. Both before and after sleep nNO levels were positively correlated with the percentage of neutrophils in nasal lavage (*r* = 0.528, *p* = 0.014; *r* = 0.702, *p* < 0.001, respectively). Additionally, before sleep nNO was also positively associated with IL-6 (*r* = 0.586, *p* = 0.005) and IL-8 (*r* = 0.520, *p* = 0.016) concentration.

**Conclusion:**

This study sustains the presence of airway inflammation in OSA patients with the increase of FeNO and nNO. The data suggests nNO might have greater value than FeNO since it positively correlated with OSA severity, and nNO is a potential bio-marker of nasal inflammation in non-smoking OSA patients.

**Electronic supplementary material:**

The online version of this article (10.1007/s11325-018-1760-1) contains supplementary material, which is available to authorized users.

## Introduction

The most important characteristic of obstructive sleep apnea (OSA) is repeated pharyngeal collapses during sleep. Local inflammation at nose, pharyngeal, and laryngeal aggravates upper airway narrowing and increases the risk of OSA. Otherwise, the mechanical damage of recurrent upper airway closure and the oxidative stress caused by intermittent oxygen desaturation induce local and systemic inflammation in OSA patients. Non-invasive methods such as exhaled gases, exhaled breath condensate, and induced sputum can be used to detect these inflammation and oxidative stress in patients with OSA [1].

Exhaled nitric oxide is regarded as a non-invasive marker of airway inflammation [2]. Several studies have evaluated fractional exhaled nitric oxide (FeNO) in OSA, but they got controversial results. In our mate-analysis, we found a high FeNO in OSA [3], but its level was still very close to the upper limit value of the health (25 ppb) which makes it impractical to be used in clinical setting. Previous study revealed a positive correlation between FeNO and nNO levels in patients with allergic rhinitis and health volunteers [4]. As nasal nitric oxide (nNO) is several times higher than FeNO [5], we hypothesize the difference of nNO level in OSA patients, and the health adults would also be much greater. Unfortunately, only six researches about nNO in patients with OSA were published, and among them, three were in pediatric OSA [6–8]; the other three were in adults with different methods of measuring nNO [9–11]. The levels of nNO in patients with OSA varied widely (from 28.4 to 610.03 ppb) in different studies, and all of these studies included less than 50 OSA patients. In the American Thoracic Society/European Respiratory Society (ATS/ERS) recommendations for standardized procedures for the measurement of nNO, the importance of velum closure was emphasized [12], to prevent loss of NO via the posterior velopharyngeal aperture or enter of lower respiratory air.

Therefore, this study had two objectives. First, we evaluated the levels of nNO in patients with OSA and healthy subjects by the ATS/ERS recommended method. Second, we investigated the relationship between nNO and FeNO and inflammatory factors in nasal lavage, in order to know whether nNO might be a noninvasive marker of upper airway inflammation in patients with OSA.

## Methods

### Study subjects

Seventy-five patients consecutively admitted to sleep center of Peking Union Medical College Hospital (PUMCH) were included. All patients were newly diagnosed OSA. Health controls were recruited from Medical Examination Center. We excluded subjects with respiratory disease including asthma, chronic obstructive pulmonary disease, infection in the last 4 weeks, rhinitis, atopic individuals, organic failure, auto-immunity diseases, treatment with oral or inhalation corticosteroids, and drugs including arginine. This study was approved by ethics committee of PUMCH, and informed consents were obtained from all participants.

### Study design

All participants admitted to sleep center of PUMCH at 8 p.m. Medical history and Epworth Sleepiness Scale (ESS) questionnaires were asked, and personal parameters were measured by well-trained researchers. Lung ventilation function including forced vital capacity (FVC) and forced expiratory volume in 1 s (FEV1) were assessed by spirometry (SuperSpiro, Micro Medical, UK).

### Exhaled nitric oxide measurement

FeNO and nNO were measured before and after sleep (9:30 to 10:00 p.m. and 6:00 to 6:30 a.m.) by NIOX MINO® (Aerocrine, Sweden). All measures were performed based on the ATS/ERS guidelines and the instruction of NIOX MINO® [13]. When FeNO was measured, subjects kept expiring at a constant flow of 50 mL/s for at least 10 s in a sitting position. When nNO was measured, subjects kept expiring with the micro-air pressure gauge ranging between 10 and 20 cmH_2_O until NIOX MINO® draw enough air from the nose (Supplement Fig. 1). Heavy exercise and smoking were inhibited 1 h before the test.

### Polysomnography

Every subject performed standard overnight polysomnography (PSG, Embla N7000, Natus Medical Incorporated, USA) from 10 pm. to 6 am. in the sleep laboratory. Electroencephalography, electrooculography, and chin electromyography recordings were recorded with surface electrodes according to standard methods. Airflow was monitored by nasal pressure. Thoracic and abdominal movements were assessed by respiratory inductive plethysmography which was recorded by belts connected to transducers. All the night recordings of pulse oxygen saturation (SpO_2_) was continuously acquired by an oximeter being attached to a finger. Electrocardiography and sleep positions also were noted. The sleep records were scored based on standardized criteria recommended by the American Academy of Sleep Medicine [14]. The sleep records were auto-scored by software, and then manually scored by a sleep laboratory expert. OSA was defined by an apnea hypopnea index (AHI) of five or more episodes per hour, associated with daytime somnolence.

### Nasal lavage

After repeatedly measured FeNO and nNO in the morning, 21 patients with OSA and ten matched health controls collected nasal lavage by an atomized nasal washing machine (Neb-nid, Flaem Nuova SPA, Italy). Rhino-Clear was filled with 6 mL natural saline and washed one nasal cavity. The other one was also washed in the same way. The collected nasal lavage samples were blindly examined within 2 h. It was filtered through gauze to remove mucus, and then centrifuged at 3000 rpm for 5 min. The supernatant was aspired for analyzing interleukin 6 (IL-6) and interleukin 8 (IL-8). The cell pellet was resuspended in 0.5 ml natural saline. The cell suspension was placed in a cytocentrifuge (Thermo Scientific Cytospin 4, Shandon, UK), and cytospin preparations were made at 800 rpm for 4 min. Cytospin slides were fixed with methanol and were stained with Wright-Giemsa by an automatic slides staining machine (Sysmex, Japan). Two investigators independently counted at least 200 cells per slide to classify cells under light microscopy and with the assistance of an electrical hemocytometer (BCC-B, Goodline Medical Technology Co. Ltd. China). IL-6 and IL-8 were measured by IMMULITE 1000 Automatic Chemiluminescence Immunoassay (Siemens Medical, USA). The intra-assay coefficients of variation (CV) were 3.5–6.2% and 3.6–3.8%, and the inter-assay CV were 5.1–7.5% and 5.2–7.4%, respectively. Both of their sensitivity were 2 pg/ml.

### Statistical analysis

Data analyses were performed using SPSS software version 21.0 (SPSS, Chicago, IL, USA) and GraphPad Prism 6.02 (GraphPad Software Inc., La Jolla, USA). Continuous variables were tested for normal distribution by Kolmogorov–Smirnov test. Results were expressed as mean ± standard deviations and as the median with interquartile range. Comparisons were made by Student’s *t* test or Mann–Whitney *U* test or one-way ANOVA being followed by a post hoc Tukey test for quantitative variables and chi-square test or Kruskal–Wallis nonparametric test for qualitative variables, as appropriate. Exhaled NO levels before and after PSG were compared using paired *t* test. Linear correlations between exhaled NO levels and clinical characteristics or other parameters were done using Pearson’s or Spearman’s method. All reported *p* values are two-sided, and *p* < 0.05 was considered to be statistically significant.

## Results

### Characteristics of study subjects

The characteristics of the participants are presented in Table 1. The OSA group was consisted of 75 male patients and was middle-aged (40.39 ± 9.58 years) and overweight with the mean body mass index (BMI) 27.73 kg/m^2^. The control group contained 30 healthy middle-aged males who were also a little bit overweight (mean BMI 24.41 kg/m^2^). All individuals had normal lung function. The median AHI was 28.1/h for OSA patients and 0.85/h for controls.

**Table 1:** Characteristics of the patients with OSA and health controls

	OSA group (*n* = 75)	Health control (*n* = 30)	*p*
Age (years)	40.39 ± 9.58	37.73 ± 10.52	0.215
Height (m)	1.74 ± 0.06	1.74 ± 0.07	0. 814
BMI (kg/m^2^)	27.73 ± 2.93	24.41 ± 2.86	< 0.001
Hypertension, *n* (%)	35 (46.7)	1 (3.3)	< 0.001
Diabetes, *n* (%)	12 (16.0)	0 (0)	0.018
Dyslipidemia, *n* (%)	3 (10)	28 (37.5)	0.005
Smokers	18 (24.0)	3 (10.0)	0.175
FEV_1_ act/pred %	103.84 ± 10.25	105.05 ± 10.46	0.589
FEV_1_/FVC %	90.21 ± 6.23	90.55 ± 6.66	0.805
ESS	11.60 ± 4.65	9.87 ± 6.00	0.116
AHI (events/h)	28.1 (13.80, 57.0)	0.85 (0.20, 1.70)	< 0.001
Nadir SpO_2_, %	81.84 ± 8.20	93.17 ± 2.38	< 0.001
Time of SpO_2_ at < 90% (min)	4.3 (0.2, 26.0)	0 (0, 0)	< 0.001

Values are presented as mean ± SD or median with interquartile range or number (percentage). AHI, apnea hypopnea index; BMI, body mass index; ESS, Epworth Sleepiness Scale; FEV_1_, forced expiratory volume in 1 s; FVC, forced vital capacity; SpO_2_, pulse oxyhaemoglobin saturation

### Exhaled NO

**Fig. 1:**
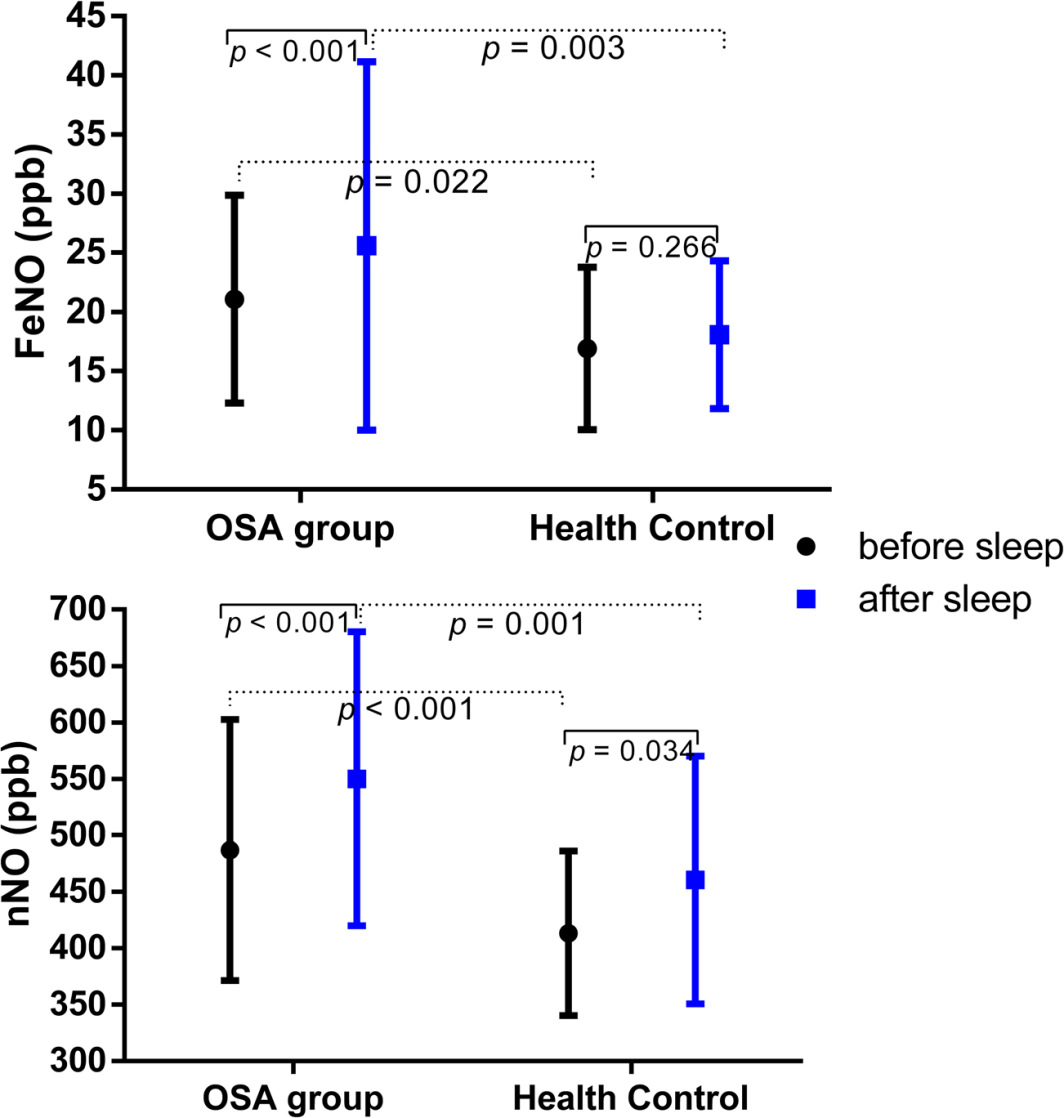
Measurement of FeNO and nNO in patients with OSA and health controls before and after sleep

As shown in Fig. 1, both before sleep and after sleep FeNO in patients with OSA were significantly higher than values in the healthy (21.08 ± 8.78 vs. 16.90 ± 6.86 ppb, *p* = 0.022, 25.57 ± 15.57 vs. 18.07 ± 6.25 ppb, *p* = 0.003, respectively). Similarly, nNO level was also higher in OSA group (before sleep nNO 487.03 ± 115.83 vs. 413.37 ± 73.10 ppb, *p* < 0.001, and after sleep nNO 550.07 ± 130.24 vs. 460.43 ± 109.77 ppb, *p* = 0.001, respectively). Interestingly, we found both FeNO and nNO levels significantly increased after sleep compared with their values before sleep (*p* < 0.001) in the OSA group, while only nNO levels increased after sleep in health controls (*p* = 0.034).

Then, we divided the OSA group into different subgroups according to BMI, AHI, hypertension, and smoking, to compare FeNO and nNO in different groups. We found in the no-smoking OSA group, all of before and sleep FeNO and nNO significantly higher than that in no-smoking health controls (Table 2), while these values were similar between smoking patients with OSA and the healthy.

**Table 2:** FeNO and nNO in no-smoking OSA, smoking OSA, and no-smoking health controls

	No-smoking OSA (*n* = 57)	Smoking OSA (*n* = 18)	Health Control (*n* = 27)	*p*
FeNO pm (ppb)	21.81 ± 7.15**	17.67 ± 9.86^#^	16.85 ± 7.21	0.001
FeNO am (ppb)	25.65 ± 8.96***	19.22 ± 10.00^#^	18.19 ± 6.38	< 0.001
nNO pm (ppb)	500.04 ± 128.76**	445.83 ± 115.73	409.26 ± 74.16	0.004
nNO am (ppb)	560.93 ± 128.76**	515.67 ± 132.58	458.26 ± 113.81	< 0.001

*Compared with health control, *p ≤ 0.017, **p ≤ 0.003, ***p≤ 0.0003

^#^Compared with no-smoking OSA, ^#^*p* ≤ 0.017, ^##^*p* ≤ 0.003, ^###^*p* ≤ 0.0003)

### Cell classification and IL-6, IL-8 in nasal lavage

The characteristics of nonsmokers (21 with OSA and 10 controls) were presented in supplement Table 1. We found the percentage of neutrophils, lymphocytes, and eosinophils were significantly higher in OSA patients compared with the healthy subjects (Table 3). Measurements of IL-6 and IL-8 also showed higher values in OSA patients compared with control subjects (Table 3).

**Table 3:** Cell classification and IL-6, IL-8 in nasal lavage of no-smoking OSA, and no-smoking health controls

	OSA (*n* = 21)	Health control (*n* = 10)	*p*
Epithelial cells (%)	79.81 ± 16.51	98.40 ± 1.35	< 0.001
Neutrophils (%)	11.50 (4.25, 26.75)	0.50 (0.00, 0.50)	< 0.001
Lymphocytes (%)	2.00 (1.00, 4.00)	0.50 (0.38, 1.25)	0.003
Eosinophils (%)	0.5 (0.0, 1.5)	0.0 (0.0, 0.0)	0.022
IL-6 (pg/mL)	2.80 (2.25, 4.30)	2.0 (2.0, 2.0)	< 0.001
IL-8 (pg/mL)	229.71 ± 175.61	69.40 ± 26.70	< 0.001

Values are presented as mean ± SD or median with interquartile range

### Correlation between after sleep exhaled NO and sleep apnea parameters and nasal lavage inflammation markers

**Fig. 2:**
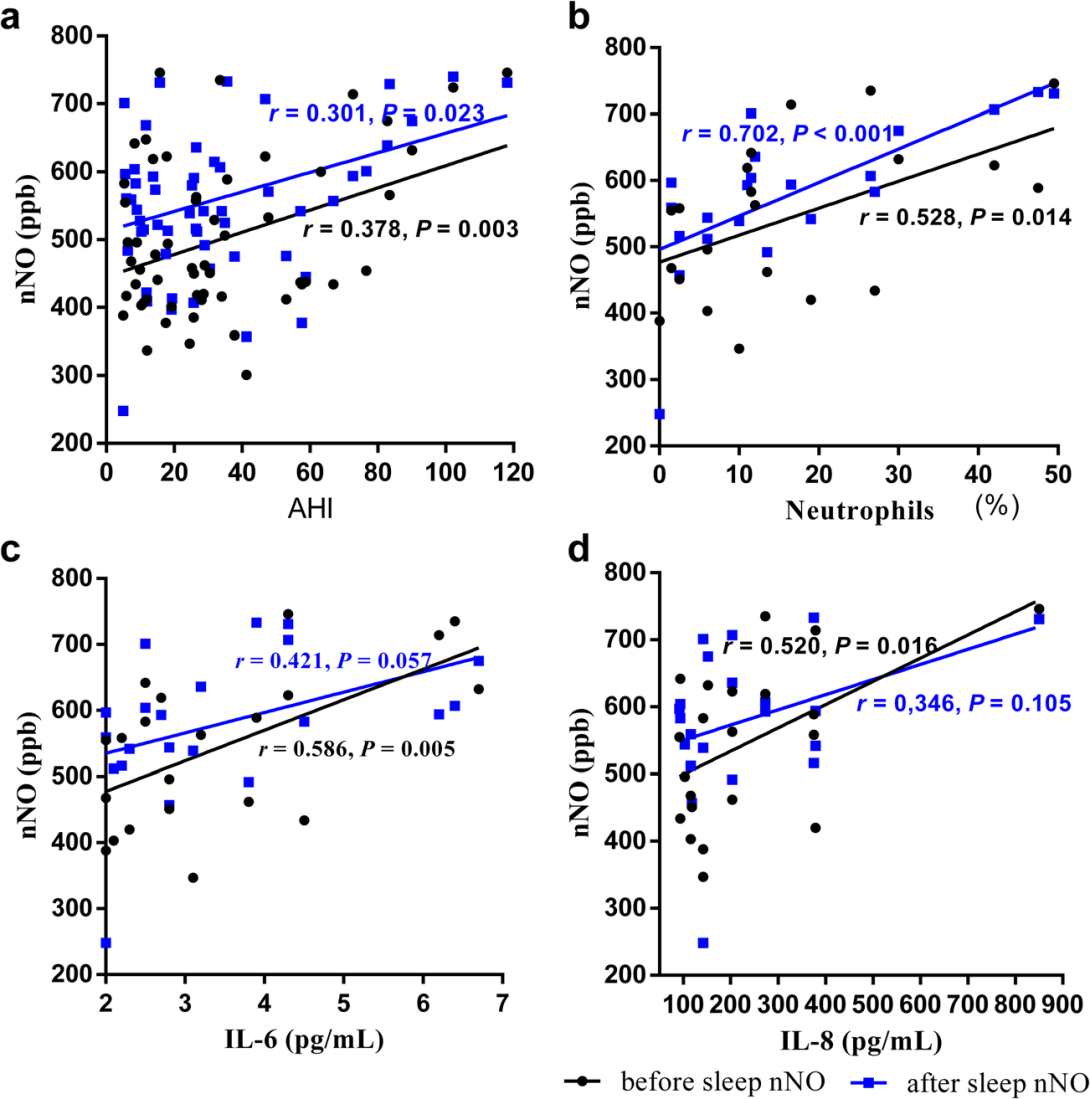
Correlation between nNO and AHI, percentage of neutrophil, IL-6, and IL-8 in nasal lavage of non-smoking OSA

In the OSA non-smokers, significant correlations were found between before sleep nNO, after sleep nNO, and AHI (*r* = 0.387, *p* = 0.003; *r* = 0.301, *p* = 0.023, respectively). Minimum SpO_2_ and total sleep time with SpO_2_ < 90% (TST SpO_2_ < 90%) were also correlated with after sleep nNO. While, the levels of FeNO before and after sleep were not significantly correlated with PSG parameters. There were direct correlation between the percentage of neutrophils and nNO (before sleep nNO *r* = 0.528, *p* = 0.014; after sleep nNO *r* = 0.702, *p* < 0.001, Fig. 2). Before sleep nNO were also positively correlated with the concentration of IL-6 (*r* = 0.582, *p* = 0.005) and IL-8 (*r* = 0.520, p = 0.016) in nasal lavage of non-smoking OSA patients. Additionally, all of neutrophils, the concentration of IL-6 and IL-8 positively associated with AHI (*r* = 0.672, *p* = 0.001; *r* = 0.676, *p* = 0.001; *r* = 0.732, *p* < 0.001, respectively).

### After sleep nasal nitric oxide for detecting non-smoking patients of OSA

**Fig. 3:**
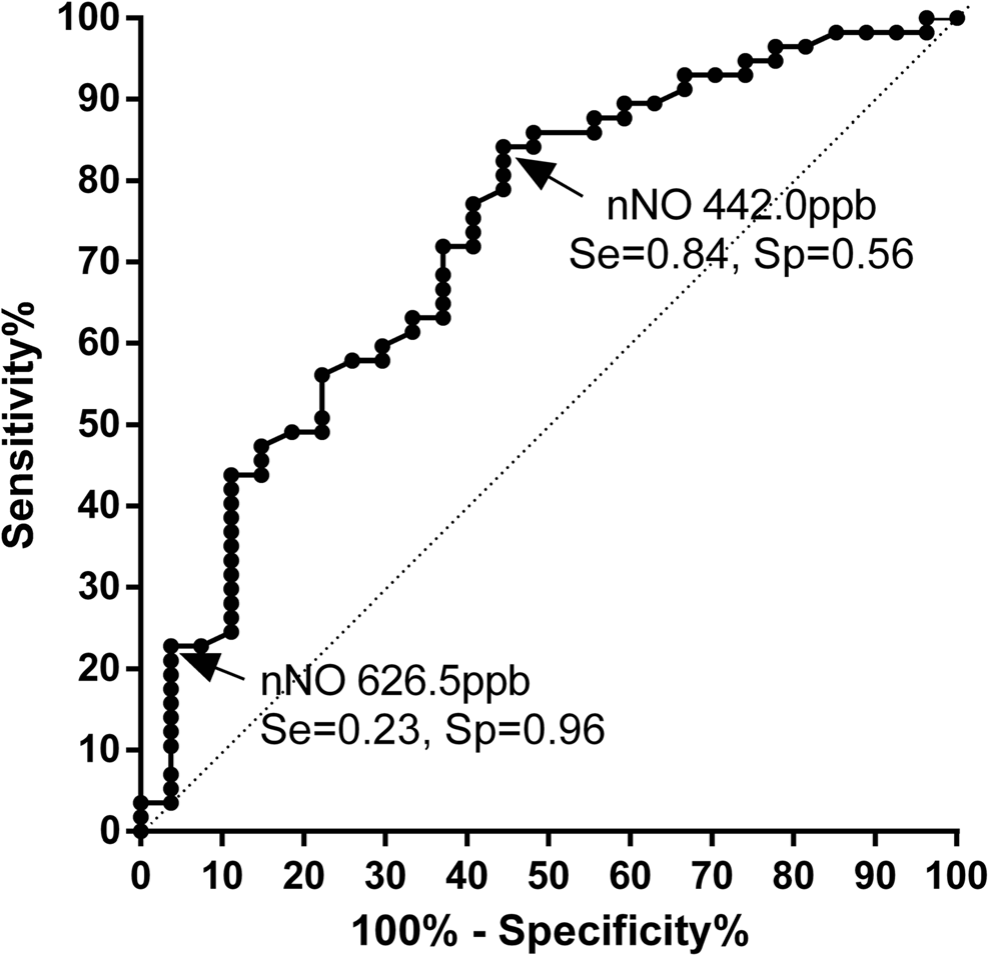
ROC curve of predicting the patients with OSA among non-smoking subjects (*n* = 84). Area under ROC curve 0.731 ± 0.060 (*p* < 0.001, 95% CI 0.613–0.848)

Because the levels of after sleep nNO were highly correlated to the severity of OSA as measured by AHI and SpO_2_, we try to used it for screening nonsmoking OSA. The area under receiver operating characteristic (ROC) curve was statistically significant (*p* < 0.001; AUC 0.731 95% CI [0.613–0.848]) (Fig. 3). The highest Youden index was at nNO level of 442 ppb with sensitivity of 84.2% and specificity of 55.6%. Another threshold of nNO at 626.5 ppb yielded higher specificity of 96.3% but lower sensitivity of 22.8%.

## Discussion

This study found that both FeNO and nNO levels were significantly higher in OSA patients than in controls. Moreover, FeNO and nNO increased after sleep in OSA patients, while nNO also slightly increased after sleep in healthy group. In smoker with OSA, the level of FeNO and nNO were lower than non-smoking OSA patients. The levels of nNO before and after sleep were closely related with sleep apnea severity as expressed by AHI, and after sleep nNO was also correlated with oxygen desaturation in non-smoking OSA. We demonstrated a neutrophilic nasal inflammation in OSA patients even when they had not shown symptoms associated with rhinitis such as runny nose or sneezing. The level of nNO was associated with nasal inflammatory marker including percentage of neutrophils, released IL-6, and IL-8.

Several studies have been published about the FeNO variation in OSA patients, but their results are controversial. The result of our study was very close to these in published meta-analysis [3] as after sleep FeNO was higher than controls by 6.50 ppb and after sleep FeNO was also increased 4.49 ppb compared with before sleep FeNO in OSA group. In non-smoking OSA patients of our study, neither before sleep FeNO, after sleep FeNO, nor ΔFeNO related to PSG parameters, which indicate FeNO, could not reflect the severity of OSA. Devouassoux et al. [15] and Depalo et al. [16] demonstrated a high neutrophil percentage and IL-8 concentration in induced sputum of untreated OSA. Depalo [16] further detected higher iNOS expression in neutrophils which was positive correlated with FeNO. These two studies shown the high FeNO come from bronchial neutrophils in OSA patients. In their studies, FeNO positively correlated with AHI, but we did not find such correlation. In the meta-analysis [3] included 688 OSA patients and 366 health controls, FeNO also did not relate to AHI. Even though OSA patients have upper airway inflammation and with a higher FeNO level, FeNO still cannot be a marker of OSA because it did not relate with OSA severity.

A major source of nNO was paranasal sinuses [17, 18] in healthy individuals. Nasal NO have important local as well as distal effects in keeping sinuses sterile, stimulating ciliary motility, and regulating pulmonary function by improving oxygen uptake and reducing pulmonary vascular resistance. Nasal NO has been used as a reliable test for primary ciliary dyskinesia [19] and a surrogate marker of nasal inflammation in allergic rhinitis [12, 20]. Up to now, only three studies on nNO concentration in adult OSA patients have been published [9–11], but their results are significantly different. In 1997, Olopade firstly measured nNO by off-line method (collect exhaled nasal gas and then test nNO concentration) [9]. With the development of technology and equipment, off-line method has been replaced by on-line way. Petrosyan [10] found a higher nNO in OSA subjects than obesity controls, and it was rectified after 1-month continuous positive airway pressure (CPAP) therapy. In his research, end-exhaled CO_2_ was measured simultaneously to make sure a satisfactory breath holding during the measurement of nNO. It got reliable results with mean nNO 610.3 ± 222.5 ppb in OSA patients. This level was a little higher than our results 550.07 ± 130.24 ppb because we used different gauge. LR2000 chemiluminenscence analyzer often get a higher result than NIOX MINO® [21]. In the recently published study of Hamada [11], nNO level was measured and calculated in a different way which lead to a much lower nNO level (from 47.9 to 76.9 ppb) in their study. In our study, we optimized the measurement of nNO by slowly exhaling orally against a resistance (micro-air pressure gauge with visual pressure) to keep velum closed. We found a higher nNO in non-smoking OSA group and the after sleep nNO was further increased than before sleep level. In non-smoking OSA patients, after sleep nNO was positively correlated with AHI and TST SpO_2_ < 90% and negatively associated to minimum SpO_2_. So nNO potentially show upper airway inflammation in OSA patients.

Nasal inflammation had been observed in OSA patients for many years [22], and the prevalence of chronic rhinitis or rhinosinusitis in OSA patients was 3.18 times of that in the healthy [23]. The most important mechanisms of upper airway inflammation in OSA was hypoxia-reflexogen-induced inflammatory cytokine release [24]. High level of inflammatory factors upregulate NF-κB pathway and increase iNOS expression further increase NO production [25]. Consistent with the animal studies, nNO aslo positively correlated with AHI, TST SpO_2_ < 90% and negatively related to minimum SpO_2_. Higher percentage of neutrophils was observed in nasal lavage of non-smoking OSA patients with no symptom rhinitis, which was similar with the result of other studies [22, 26]. Cellular classification of induced sputum in OSA patients also found a higher percentage of neutrophils [15, 16]. Depalo measured iNOS expression in sputum cells by immunocytochemistry and showed higher iNOS expression in neutrophils of OSA patients. This indicated the origin of increased exhaled NO level. Similarly, the high percentage of neutrophils in nasal lavage of OSA was positively correlated with nNO.

Studies showed that the concentration of IL-6 and IL-8 increased in exhaled breath condensate and induced sputum [27–29]. We observed an increase of IL-6 and IL-8 in nasal lavage of non-smoking OSA. All of IL-6, IL-8, and percentage of neutrophils positively correlated with AHI, which indicated they were associated with OSA severity. Researches have shown IL-6 and IL-8 may mediate nasal inflammation in OSA patients. Hypoxia can activate NF-κB pathway and prompt release of IL-6 [30] and IL-8 [31] to evoke inflammation. For the best of our knowledge, our study firstly observed the positive correlation between nNO and percentage of neutrophils, IL-6, IL-8 in nasal lavage, and AHI, which indicates that nNO was a marker of nasal inflammation and severity of OSA. However, in our study, the expression of iNOS in neutrophils was not tested, and the effect of CPAP therapy on nNO was not followed.

As nNO level related to OSA severity, we made a ROC curve to primarily evaluate whether nNO could be used in clinical screening of OSA. We suggest when the after sleep nNO higher than 626 ppb, PSG should be recommended to confirm OSA diagnosis. This cutoff value of nNO has a high specificity but low sensitivity to detect OSA. As there are still no effective questionnaires or models to screen OSA so far, further researches are needed to combine nNO with other methods to improve the accuracy in predicting OSA [28]. However, only few studies with small samples about nNO are available at present, and the normal range of nNO still need to be worked out. So, the cutoff value need further verification in large population. Therefore, a great distance exists from the clinical use of nNO.

Moreover, the percentage of lymph cell and eosinophil in nasal lavage of OSA patients was higher than that of health controls. But their percentages were too small to meet the criterion of allergic rhinitis; we speculate they were also a performance of nasal inflammation. In pharyngeal lavage, lymph especially CD4+ T cells, IL-6, and IL-8 were higher in OSA patients than in snorers or healthy controls, and they were also positively correlated with AHI. All of them decreased significantly after 1 year of therapy with CPAP or surgery [32].

Nasal mucosa swelling and cavity obstruction, being caused by nasal inflammation, can increase the severity of OSA. A lot of OSA patients have subclinical nasal inflammation without common symptoms, so it is very hard to be diagnosed. But this upper airway inflammation may be closely connected with noncompliance with nasal CPAP therapy [26]. So, further studies are still needed to detect the relationship between nNO and nasal inflammation, CPAP complication, and the effect of CPAP, in order to know whether nNO can be a predictor of CPAP complication or follow-up marker of CPAP effects.

We found a lower FeNO and nNO level in smoking OSA than non-smoking OSA patients, inconsistent with the previous studies which also indicate a lower FeNO in smokers [33, 34]. Because smoking can inhibit the expression of iNOS to reduce production and activity of NO [33, 35], and the harmful gases such as free radicals and superoxide in smoking have chemical reaction with NO to form nitrite amine. Smoking and OSA have opposite effects on FeNO and nNO level, so the concentration of FeNO and nNO in smoking OSA were close to the normal range.

Interestingly, we found that after sleep nNO significantly increased than before sleep nNO in OSA group and health controls in our study. The increase in OSA patients was a little more than that of control subjects, but still did not reach a significant difference. It is not clear whether NO output has circadian variation. Dias [36] observed the concentration of exhaled NO increased from early morning to midday to late afternoon, which suggest a possible circadian relationship. But O’Hacken et al. [37] measured exhaled NO once an hour and found no significant temporal pattern over a 24-h period. Chatkin et al. [38] showed nNO concentration was negatively associated with nasal air-flow, and O’Hearn found increase in exhaled NO concentration after daytime awake voluntary hypoventilation (6.8 ± 1.4 L/min) [37]. So, the confounder trans-nasal minute ventilation need to be checked before determining whether nNO can be used to reflect upper-airway inflammation in OSA patients.

### Limitations

There were some limitations in this study. Firstly, the sample size was small and only include adult male; further studies are needed to confirm whether the results are applicable to female. Secondly, we did not perform nasal sinus imageological examination and bronchial provocation test; the exclusion of rhinitis, nasosinusitis, and asthma was mainly depended on history and clinical manifestation. Thirdly, nasal minute ventilation was not measured, so it was unknown whether nNO levels affected by nasal ventilation or not. Fourthly, nNO has not been widely used in clinical practice and without normal value range in China. But, the result of healthy controls in our study was very similar with that of other research (446.76 ± 133.63 ppb) [13].

## Conclusion

Patients of OSA have non-symptom neutrophic rhinitis, with higher nNO concentration. Nasal NO positively correlated with OSA severity and rhinitis, suggest nNO is a promising marker of nasal inflammation in non-smoking OSA. Further studies are needed to better understand the effect of CPAP therapy on nNO and the relationship between nNO and CPAP compliance.

## Electronic supplementary material

ESM 1 (DOCX 17 kb)

Figure 1 A method for on-line detection of nasal nitric oxide (nNO) in human subjects using a micro-air pressure gauge to keep a pressure varying from 10 cmH_2_O to 20 cmH_2_O to maintain velum closure. (PNG 597 kb)

High Resolution Image (TIF 1048 kb)

## Abbreviations

AHI: Apnea hypopnea index
ATS/ERS: American Thoracic Society/European Respiratory Society
BMI: Body mass index
CPAP: Continuous positive airway pressure
CV: Coefficients of variation
ESS: Epworth Sleepiness Scale
FeNO: Fractional exhaled nitric oxide
FEV1: Forced expiratory volume in 1 s
FVC: Forced vital capacity
IL: Interleukin
iNOS: Inducible nitric oxide synthase
nNO: Nasal nitric oxide
OSA: Obstructive sleep apnea
PSG: Polysomnography
PUMCH: Peking Union Medical College Hospital
SpO_2_: Pulse arterial oxygen saturation
TST SpO_2_ < 90%: Total sleep time with SpO_2_ < 90%
ROC: Receiver operating characteristic curve
