# Supplementary material for: Measurement of fractional exhaled nitric oxide and nasal nitric oxide in male patients with obstructive sleep apnea
Source: Sleep Breath. 2018 Dec 12;23(3):785–93. doi: 10.1007/s11325-018-1760-1 (PMC6700235; doi:10.1007/s11325-018-1760-1)
Supplement: Supplementary file 1 — (DOCX 17 kb) [file 11325_2018_1760_MOESM1_ESM.docx]

**Supplement Table 1**：Characteristics of the patients with OSA and health controls performed nasal lavage

|  | OSA group (n = 21) | Health Control (n = 10) | *P* |
| --- | --- | --- | --- |
| Age（years） | 39.33 ± 11.31 | 36.10 ± 8.62 | 0.431 |
| BMI（kg/m^2^） | 26.41 ± 2.98 | 24.95 ± 2.27 | 0.182 |
| Hypertension,n (%) | 11 (52.4) | 1 (10.0) | 0.046 |
| Diabetes, n (%) | 3 (14.3) | 0 (0) | 0.533 |
| Dyslipidemia, n (%) | 7 (33.3) | 1 (10.0) | 0.066 |
| FEV_1_ act/pred % | 102.86 ± 9.71 | 105.00 ± 11.51 | 0.593 |
| FEV_1_ / FVC % | 88.95 ± 5.94 | 89.46 ± 7.95 | 0.843 |
| ESS | 10.43 ± 3.85 | 8.00 ± 4.76 | 0.139 |
| AHI (events / h) | 30.28 ± 29.95 | 0.73 ± 1.20 | < 0.001 |
| Nadir SpO_2_, % | 83.71 ± 7.10 | 93.60 ± 1.71 | < 0.001 |
| Time of SpO_2_ at < 90 % (min) | 2.7 (0.15, 12.48) | 0 (0,0) | < 0.001 |

Values are presented as mean ± SD or median with interquartile range or number (percentage). AHI, apnea hypopnea index; BMI, body mass index; ESS, Epworth Sleepiness Scale; FEV_1_, forced expiratory volume in 1 second; FVC, forced vital capacity; SpO_2_ , pulse oxyhaemoglobin saturation.
