# Supplementary figures and images for: Measurement of fractional exhaled nitric oxide and nasal nitric oxide in male patients with obstructive sleep apnea
Source: Sleep Breath. 2018 Dec 12;23(3):785–93. doi: 10.1007/s11325-018-1760-1 (PMC6700235; doi:10.1007/s11325-018-1760-1)

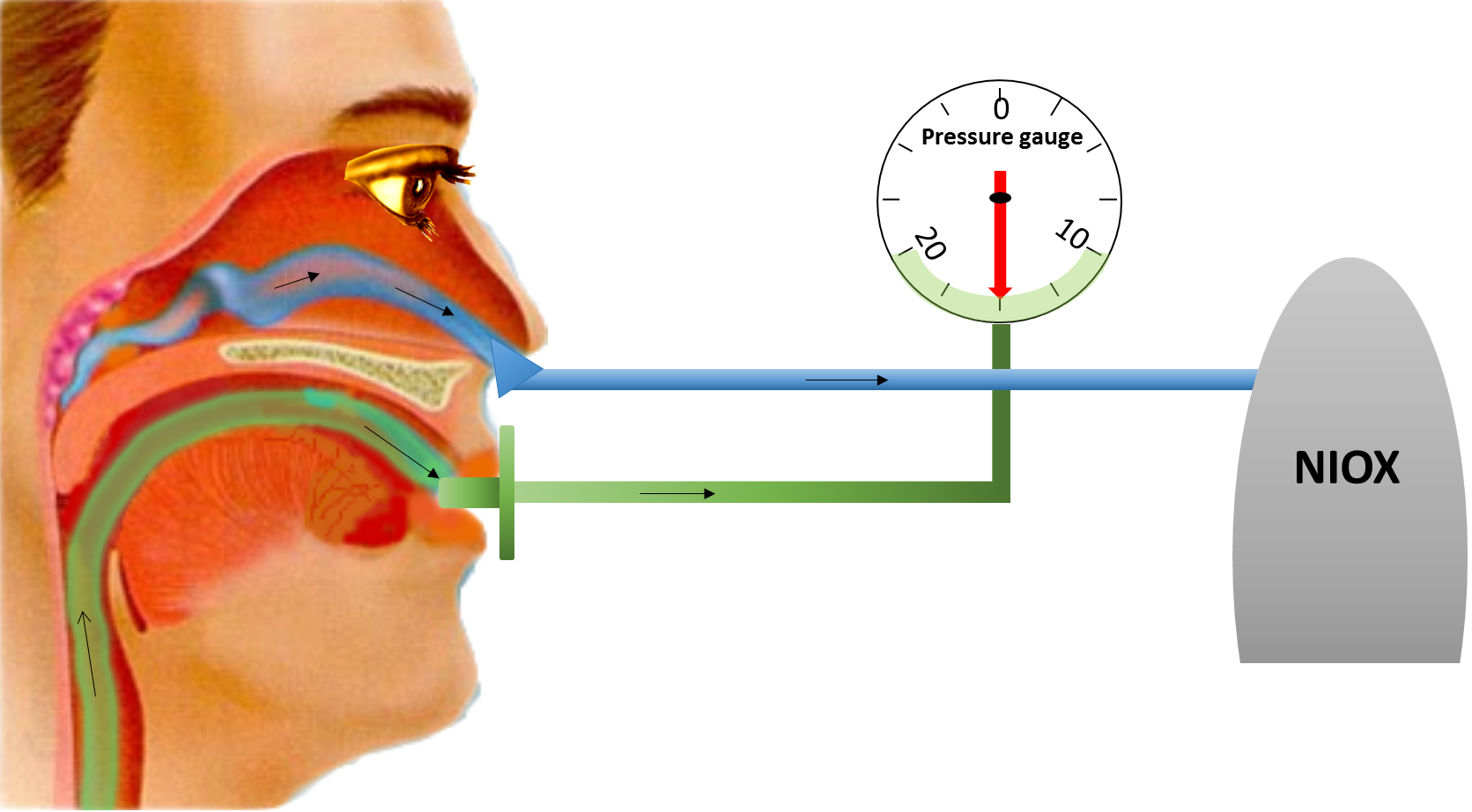

Supplement: Supplementary file 2 — A method for on-line detection of nasal nitric oxide (nNO) in human subjects using a micro-air pressure gauge to keep a pressure varying from 10 cmH2O to 20 cmH2O to maintain velum closure. (PNG 597 kb) [file 11325_2018_1760_Fig4_ESM.png]

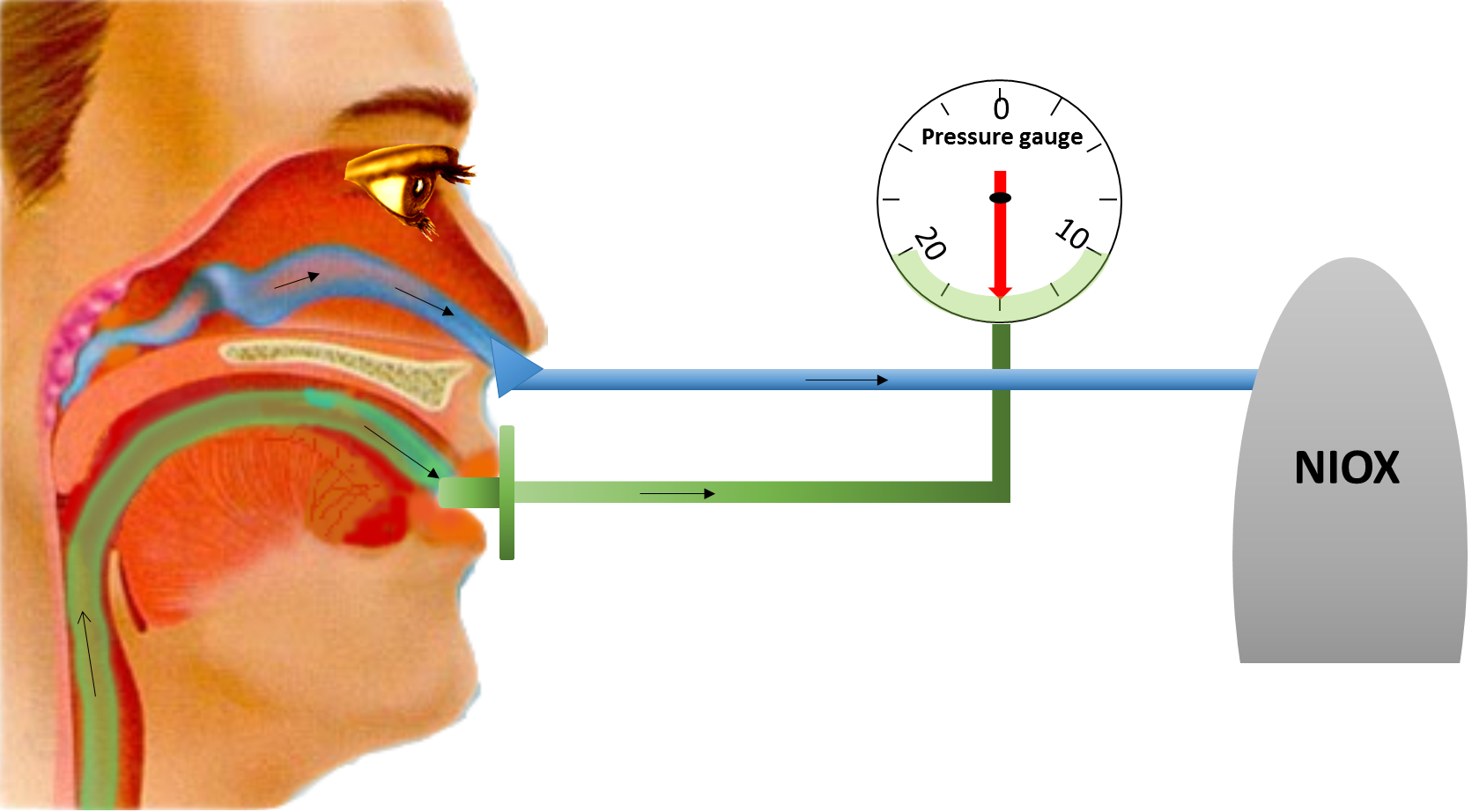

Supplement: Supplementary file 3 — High Resolution Image (TIF 1048 kb) [file 11325_2018_1760_MOESM2_ESM.tif]
